# Complete mitochondrial genome of *Halocynthia hilgendorfi ritteri* (Pyuridae) from Korea

**DOI:** 10.1080/23802359.2020.1831987

**Published:** 2020-10-27

**Authors:** Kang-Rae Kim, Moo-Sang Kim, Yong Hwi Kim, Jong Yeon Park, In-Chul Bang

**Affiliations:** a Department of Life Science & Biotechnology, Soonchunhyang University, Asan, Republic of Korea; b Department of Bioinformatics, The Moagen, Daejeon, Republic of Korea

**Keywords:** Complete mitochondria genome, Pyuridae, *Halocynthia hilgendorfi ritteri*, phylogenetic analysis

## Abstract

*Halocynthia hilgendorfi ritteri* is an ascidian distributed on the coast of Geoje Island in Korea and found on rocks. The mitochondrial genome of *Halocynthia hilgendorfi ritteri* consists of 15,181 bp with 13 protein-coding genes, 2 ribosomal RNAs, 23 transfer RNA genes. The overall base composition of the complete genome is 22.94% A, 43.32% T, 25.72% G, and 8.02% C, with a high A + T content of 66.26%.

*Halocynthia hilgendorfi ritteri* is a subspecies of *Halocynthia hilgendorfi* that belongs to the family Pyuridae, order Pleurogona. It is native to Asia and occurs off the coast of Jeju Island, Korea (Oka 1906; Tokioka 1959; Choi, Lee, et al. 2004). *Halocynthia hilgendorfi ritteri* is found mainly on rock surfaces and its shape and color are similar to those of the surrounding rocks (Choi, Kim, et al. 2004). *Halocynthia hilgendorfi ritteri*, an important fishery resource in Korea, is widely consumed and popular. The species in the genus *Halocynthia* are distinct when alive, but are difficult to distinguish when processed. Therefore, we would like to report the complete mitogenome sequence of *H. hilgendorfi ritteri* for developing a marker to identify this subspecies.

*Halocynthia hilgendorfi ritteri* were collected from Geoje Island (34°53′N, 128°37′E) and DNA was extracted using a DNeasy Blood and Tissue Kit (QIAGEN, Hilden, Germany). The genomic DNA is preserved at Soonchunhyang University, Republic of Korea. A complete mitochondrial genome was obtained according to the MGISEQ-2000 protocol (MGI, China) using an MGI Easy DNA Library Prep Kit (MGI, Wuhan, China). The *H. hilgendorfi ritteri* sequence has been registered at NCBI GenBank (Accession no. MT811760).

The complete mitochondrial genome of *H. hilgendorfi ritteri* consists of 15,181 bp, with 13 protein-coding genes (PCGs), 2 ribosomal RNA genes (rRNAs), and 23 transfer RNA genes (tRNAs). The *CO1*, *Cytb*, *ND2*, *ND4L*, and *ATP6* genes include the ATG start codon; the remaining eight PCGs start with GTG. Six PCGs (*CO3*, *Cytb*, *ND2*, *ND4L*, *ND5*, and *ND6*) terminate with TAG, a typical stop codon and seven PCGs (*CO1*, *CO2*, *ND1*, *ND3*, *ND4*, *ATP6*, and *ATP8*) end with a complete TAA. All the genes are encoded on the heavy strand, except for the two tRNA genes (*Arg* and *Ser1*) and 12 PCGs (*ATP6*, *ATP8*, *CO1*, *CO3*, *Cytb*, *ND1*, *ND2*, *ND3*, *ND4*, *ND4L*, *ND5*, and *ND6*). The overall base composition of the *H. hilgendorfi ritteri* genome is 22.94% A, 43.32% T, 25.72% G, and 8.02% C, with a high A + T content of 66.26%. rRNA consists of small rRNA (672 bp) and large rRNA (1126 bp).

We used the maximum likelihood method to construct a phylogenetic tree based on the complete CO1 of *Halocynthia* species and included other Pyuridae family sequences as an outgroup (Figure 1). This method identified *H. hilgendorfi ritteri* as being closely related to *Halocynthia spinosa*.

**Figure 1.**
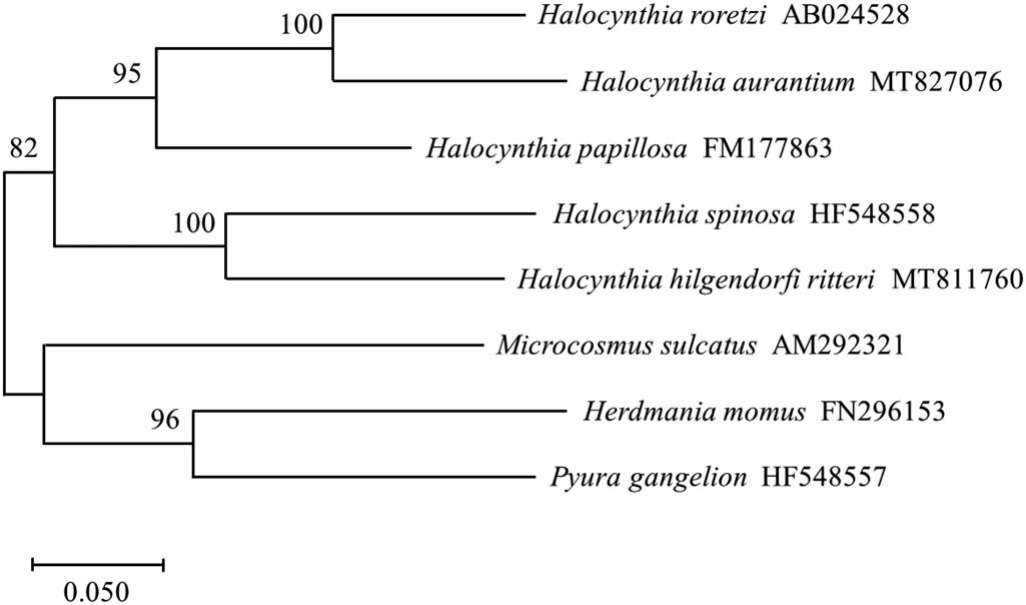
Molecular phylogenetic tree of *cytochrome oxidase subunit 1* (*CO1*) based on Pyuridae species. The relationships were identified using the maximum likelihood method and 1000 bootstrap replicates.

## Data Availability

The data that support the findings of this study are openly available in GenBank of NCBI at https://www.ncbi.nlm.nih.gov/nuccore/MT811760, reference number MT811760.
